# Interleukin-2 gene transfer into human transitional cell carcinoma of the urinary bladder

**DOI:** 10.1038/sj.bjc.6690124

**Published:** 1999-02

**Authors:** M Milella, J Jacobelli, F Cavallo, A Guarini, F Velotti, L Frati, R Foà, G Forni, A Santoni

**Affiliations:** Department of Experimental Medicine and Pathology, University of Rome ‘La Sapienza’, viale Regina Elena 324, Rome, 00161, Italy; Consiglio Nazionale delle Ricerche-Centro Immunogenetica ed Oncologia Sperimentale, via Santena 19, Turin, 10126, Italy; Department of Biomedical Sciences and Human Oncology, University of Turin, via Genova 3, Turin, 10126, Italy; Department of Scienze Ambientali, University of Tuscia, Viterbo, Italy

**Keywords:** human, interleukin-2, transitional bladder cancer, gene therapy, immunotherapy

## Abstract

Transitional cell carcinoma of the bladder is one of the human cancers most responsive to immunotherapy, and local interleukin-2 (IL-2) production appears to be an important requirement for immunotherapy to be effective. In this study, we engineered two human bladder cancer cell lines (RT112 and EJ) to constitutively release human IL-2 by retroviral vector-mediated gene transfer. Following infection and selection, stable and consistent production of biologically active IL-2 was demonstrated at both the mRNA and the protein level. Morphology, in vitro growth rate and proliferation, as well as other cytokine gene mRNA or membrane adhesion receptor expression, were not altered in IL-2 transduced cells as compared to their parental or control vector-infected counterparts. Moreover, IL-2 engineered cells lost their tumorigenicity into *nu/nu* mice and the mechanism of rejection appeared to involve multiple host effector cell populations, among which a prominent role was played by neutrophils and radiosensitive cells. These findings may offer support to the development of an IL-2-based gene therapy approach to human bladder cancer. *1999 Cancer Research Campaign*


*British Journal of Cancer* (1999) **79**(5/6), 770–779
© 1999 Cancer Research Campaign
Article no. bjoc.1998.0124

### Interleukin-2 gene transfer into human transitional cell carcinoma of the urinary bladder

**M Milella[1], J Jacobelli[1], F Cavallo[2], A Guarini[3], F Velotti[4], L Frati[1], R Foà[3], G Forni[2] and A Santoni[1]**

[1]Department of Experimental Medicine and Pathology, University of Rome 'La Sapienza', viale Regina Elena 324, 00161 Rome, Italy; [2]Consiglio Nazionale delle Ricerche-Centro Immunogenetica ed Oncologia Sperimentale, via Santena 19, 10126 Turin, Italy; [3]Department of Biomedical Sciences and Human Oncology, University of Turin, via Genova 3, 10126 Turin, Italy; [4]Department of Scienze Ambientali, University of Tuscia, Viterbo, Italy

**Summary** Transitional cell carcinoma of the bladder is one of the human cancers most responsive to immunotherapy, and local interleukin-2 (IL-2) production appears to be an important requirement for immunotherapy to be effective. In this study, we engineered two human bladder cancer cell lines (RT112 and EJ) to constitutively release human IL-2 by retroviral vector-mediated gene transfer. Following infection and selection, stable and consistent production of biologically active IL-2 was demonstrated at both the mRNA and the protein level. Morphology, in vitro growth rate and proliferation, as well as other cytokine gene mRNA or membrane adhesion receptor expression, were not altered in IL-2 transduced cells as compared to their parental or control vector-infected counterparts. Moreover, IL-2 engineered cells lost their tumorigenicity into *nu/nu* mice and the mechanism of rejection appeared to involve multiple host effector cell populations, among which a prominent role was played by neutrophils and radiosensitive cells. These findings may offer support to the development of an IL-2-based gene therapy approach to human bladder cancer.

**Keywords:** human; interleukin-2; transitional bladder cancer; gene therapy; immunotherapy

Although cytokine immunotherapy has been regarded for many years as one of the most promising strategies to achieve long-term control of solid tumours, clinical results have been to a large extent disappointing. This is due both to the high toxicity of systemically administered cytokines and to the heterogeneity and unpredictability of clinical responses. Recent advances suggest that the ability of interleukin-2 (IL-2) to generate and/or amplify an effective immune response relies on its intratumoral, rather than systemic, availability (Forni et al, 1988; Sitkovsky and Paul, 1988). IL-2 delivery at the tumour site is effective in providing a 'local help' for the induction of anti-tumour cytotoxic T lymphocytes (CTLs) (Fearon et al, 1990). By acting in a paracrine fashion, intratumoral IL-2 bypasses the requirement for T-helper cells that are paralysed by the lack of adequate costimulation or by the presence of tumour-derived immunosuppressive factors (Fearon et al, 1990; Guarini et al, 1997). Moreover, local IL-2 delivery circumvents the toxicity elicited by the systemic administration of high cytokine doses.

Effective gene-engineering techniques make cancer cells capable of releasing a cytokine within a growing tumour. Over the last few years, the successful insertion of several cytokine genes into murine and human cancer cells has been documented. Studies in mice have shown that IL-2 release by the engineered neoplastic cell leads to tumour rejection and specific CTL generation, and confers an immunological memory against subsequent challenges of the parental tumour (Fearon et al, 1990; Musiani et al, 1997). Moreover, vaccination with IL-2 transduced tumour cells has proven effective in the 'cure' of established parental tumours (Musiani et al, 1997). Human cancer cells of different origin (melanoma, renal cell carcinoma, lung carcinoma, acute leukaemia) have also been successfully transduced with different cytokine genes (Gansbacher et al, 1992; Gastl et al, 1992; Alosco et al, 1993; Cignetti et al, 1994; Hathorn et al, 1994; Guarini et al, 1995, 1996, 1997; Meazza et al, 1996; Stein et al, 1996), and therapeutic vaccination trials with IL-2 engineered tumour cell lines have been activated in advanced melanoma and renal cell carcinoma patients (Dranoff and Mulligan, 1995).

Transitional cell carcinoma of the bladder is the third most common cancer in males in Western countries. Superficial bladder cancer is responsive to intravesical treatment with bacillus Calmette–Guérin (BCG), which potentiates host immune responses and induces significant urinary IL-2 levels (Lum and Torti, 1995). Regional (intra-arterial or intravesical) recombinant IL-2 (rIL-2) administration is also effective in inducing tumour regression, by means of increasing an inflammatory reaction and activating an immune response at the tumour site (Velotti et al, 1994; Lum and Torti, 1995). Studies in a mouse tumour model of bladder cancer have recently shown that irradiated IL-2 gene-modified MBT-2 tumour cells cure a substantial proportion of mice from a significant burden of parental tumour implanted orthotopically into the bladder wall (Connor et al, 1993). Cured mice are capable of rejecting a subsequent challenge with a highly tumorigenic dose of parental unmodified MBT-2 cells, thus suggesting the induction of a protective immunological memory. These clinical and experimental findings suggest that IL-2 gene therapy may be a very effective therapeutic approach for bladder cancer. Surprisingly, no data have been reported on human bladder carcinoma cells, so far. Therefore, in this preclinical study we tested the feasibility of transducing human bladder cancer cells with the human IL-2 gene and analysed the phenotypic and functional characteristics of the transduced cells.

*Received 17 November 1997*
*Revised 16 April 1998*
*Accepted 28 May 1998*

*Correspondence to:* A Santoni

### MATERIALS AND METHODS

### Cell lines

The human transitional bladder carcinoma cell lines RT112 and EJ (also known as MGH-U1) were kindly provided by Dr Prescott (Department of Surgery/Urology, Western General Hospital, Edinburgh, UK). Both cell lines were cultured in Roswell Park Memorial Institute (RPMI)-1640 medium (Gibco, Paisley, UK) supplemented with 10% fetal calf serum (FCS, Gibco), 50 U/ml penicillin, 50 μg/ml streptomycin and 2 mM glutamine (Flow Laboratories, Irvine, UK).

### IL-2 gene transduction

IL-2 cDNA cloned from human peripheral blood lymphocytes was inserted into the Moloney murine leukaemia virus-derived retroviral vector LXSN containing the selectable marker NeoR, and producer cells were obtained by transfection as previously described (Dusty Miller and Rosman, 1989; Melani et al, 1994). RT112 or EJ cells ($10^6$) were exposed to undiluted viral supernatant for 3 h in the presence of 8 μg $ml^{-1}$ of polybrene, and selected in 0.8 mg $ml^{-1}$ G418 (Sigma Chemical Co., St Louis, MO, USA). RT112 or EJ cells infected with the LXSN vector were used as a transfection control in all experiments.

### Proliferation assay

Five thousand cells/well were seeded in flat bottom 96-well microplates and cultured in triplicates under different conditions (see Figure 2). Cells were labelled with 74 kBq of [$^3$H]TdR (methyl-$^3$H-thymidine, 185 GBq mmol, 5 Ci $mmol^{-1}$, Amersham Life Sciences, Milano, Italy)/well, harvested 18 h later, and [$^3$H]TdR uptake was determined by liquid scintillation spectrometry and expressed as total counts per minute (cpm).

To measure released IL-2 biological activity, $2.5 \times 10^5$ infected cells were seeded in 3.5 cm plates, cultured for 48 h, and the supernatants were then harvested. IL-2 biological activity in the supernatants was assessed by measuring the growth of either an IL-2-dependent murine T-cell line (CTLL) or a preparation of human phytohaemagglutinin (PHA)-stimulated T-cell blasts from healthy donors in a proliferation assay. IL-2 activity in the supernatants was expressed as International Units (IU): the titer that gave 50% of maximal [$^3$H]TdR incorporation was determined by a probit analysis computer program (Sette et al, 1986). Our laboratory standard was calibrated against that of the Biological Response Modifier Program (National Cancer Institute, Bethesda, MD, USA) reference reagent human IL-2 (lot ISDP-841).

### Cytokine mRNA expression

Total RNA was extracted by the method of Chomczynski and Sacchi (1987). Reverse transcription (RT) was performed on 5 μg of total RNA; the reaction mixture included: 1 μg oligo-dT, 1 mM deoxyribonucleotides (dNTPs), 5 μl of the 10 × RT buffer [200 mM Tris-HCl pH 8.8, 500 mM KCl, 1 mg $ml^{-1}$ bovine serum albumin (BSA)], 25 mM magnesium chloride ($MgCl_2$), 40 U Rnasin and 100 U reverse transcriptase Superscript Rnase H (Gibco). Polymerase chain reaction (PCR) amplification was performed in a final volume of 50 μl containing 1 μl of cDNA, 2.5 μl of each primer at a concentration of 20 μM, 3 μl of 2.5 μM dNTPs, 3 μl of 25 mM $MgCl_2$, 5 μl of 10 × PCR reaction buffer (200 mM Tris-HCl pH 8.4, 500 mM KCl) and 0.5 μl of 5 U $ml^{-1}$ *Taq* polymerase (Gibco). The following temperature conditions were used for the reactions: 2 min initial denaturation at 94°C, then 32 cycles of: 1 min denaturation at 94°C; annealing at 60°C (IL-2), 55°C (IL-6), 63°C [tumour necrosis factor-α (TNF-α) and β2-microglobulin ($β_2$m), or 59°C (IL-4, transforming growth factor-β (TGF-β) and granulocyte-macrophage colony-stimulating factor (GM-CSF)]; synthesis for 1 min at 72°C. Specific primers were synthesized on the basis of published sequences. PCR products were electrophoresed on a 2% agarose gel in Tris–borate–EDTA buffer. Gels were then stained with ethidium bromide and photographed.

### Immunofluorescence and cytofluorimetric analysis

The following commercially available mouse monoclonal antibodies (mAbs) were used: anti-α1 (T Cell Sciences Inc., Cambridge, MA, USA); anti-α2, anti-α4 and anti-α5 (Telios Pharmaceutical Inc., San Diego, CA, USA); anti-αv, anti-β3 and anti-ICAM-1 (Immunotech, Marseille, France); anti-β1 4B4 clone (Coulter Immunology, Hialeah, FL, USA); anti-CD44 standard form and variants v4, v6 and v9 (Bender Medsystem, Wien, Austria). The anti-α2 mAb 5E8 was kindly provided by Dr Bankert (Rosewell Park Cancer Institute, Buffalo, NY, USA); Dr F Sanchez-Madrid (Hospital de la Princesa, Madrid, Spain) generously provided the anti-α4 mAb HP2/1; the anti-α5 Mab16 was a kind gift of Dr KM Yamada (National Institutes of Health, Bethesda, MD, USA); the rat anti-α6 mAb GoH3 was generously provided by Dr A Sonnenberg (Netherlands Red Cross Laboratory, Amsterdam, The Netherlands); the rat anti-β4 mAb 439-9B was a kind gift of Dr A Sacchi (Regina Elena Cancer Institute, Rome, Italy); A1A5 anti-β1 mAb was kindly provided by Dr M Hemler (Dana-Farber Cancer Institute, Boston, MA, USA); anti-α3 mAb M-KID2 was a kind gift of Dr A Bartolazzi (Regina Elena Cancer Institute, Rome, Italy). Human leucocyte antigen (HLA) class I expression was analysed using the W6/32 mAb, generously provided by Dr G Trinchieri (Wistar Institute, Philadelphia, PA, USA).

One million cells were stained with the relative primary antibody for 30 min at 4°C; after washing, cells were incubated with a secondary fluorescein isothiocyanate (FITC)-conjugated antibody for 30 min at 4°C. The relative fluorescence intensity was determined with a FACScan (Becton Dickinson, Mountain View, CA, USA) and expressed in arbitrary units on a logarithmic scale.

### Tumorigenicity in *nu/nu* mice

Six-week-old female *nu*/*nu* mice of CD1 background (Charles River Laboratories, Calco, Italy) were maintained under strict pathogen-free conditions, receiving sterilized pellets and water ad libitum; their care was in accordance with established international guidelines. After subcutaneous (s.c.) injection of transduced cells ($10^6$, $5 \times 10^6$, $10^7$) into the left groin region, mice were monitored twice weekly to measure tumour growth. Mice with neoplastic masses > 10 mm were sacrificed for humane reasons. Immunodepletion was performed as follows: starting 2 days before tumour challenge and 4 h after and then at biweekly intervals, mice received intraperitoneal (i.p.) injections of 0.2 ml of phosphate buffered saline containing a 1/20 dilution of anti-asialoganglioside GM1 rabbit antiserum (Wako Chemicals, Dusseldorf, Germany), 100 μg of anti-polymorphonuclear leucocyte mAb (RB6-8C5

© Cancer Research Campaign 1999

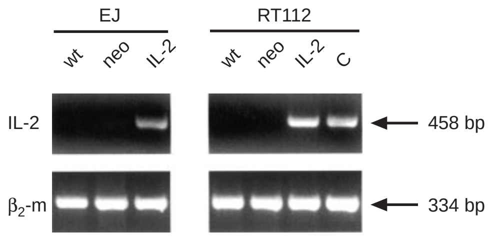

| Cell line | IL-2 biological activity (IU/$10^6$ cells/48 h) |
|---|---|
| RT112–wt | <0.05 |
| RT112–neo | <0.05 |
| RT112–IL-2 | 194 |
| EJ–wt | <0.05 |
| EJ–neo | <0.05 |
| EJ–IL-2 | 113 |

**Figure 1** IL-2 mRNA expression by transduced RT112 and EJ human bladder cancer cell lines. Parental (wt), control-vector (neo), and IL-2 (IL-2)-transduced RT112 and EJ cells were analysed for IL-2 mRNA by RT-PCR (top panel). Total cellular RNA was extracted and reverse transcription, as well as cDNA amplification, was performed as described in Materials and methods. RNAs from T-cell blasts obtained by 72-h PHA stimulation of human PBMC from healthy donors were used as positive controls (C). The $\beta_2$-m amplification represents the positive control of cDNA transcription. IL-2 biological activity assay (bottom panel) was performed as described in Materials and methods. IL-2 values are expressed in IU (as evaluated by probit computer analysis of the titration curve) and represent the mean of at least three different determinations over a period of at least 6 months hybridoma, kindly provided by Dr RL Coffman, DNAX Inc., Palo Alto, CA, USA) or normal rat immunoglobulins purified from serum or ascitic fluid by passage through an anionic exchange column (DE 52, Whatman Ltd, Maidstone, UK). Cytofluorimetric analysis of the blood and spleen cells from mice receiving these antibodies showed that target leucocytes were selectively decreased to less than 1/5000 of peripheral blood leucocytes during treatment. Selective depletion of radiosensitive cells was obtained by exposing mice to 4.5 Gy total body irradiation from a $^{137}$Cs source.

### RESULTS

### Transduction of human bladder carcinoma cell lines with the IL-2 gene

The bladder carcinoma cell lines RT112 and EJ, derived from two human transitional cell carcinomas with different histopathological grade (G2 and G3 for RT112 and EJ, respectively), were used as targets of retroviral-mediated IL-2 gene transduction. RT112 and EJ cells exhibit different in vitro growth rate and colony-forming efficiency, as well as a distinct pattern of integrin expression and adhesive ability to extracellular matrix (ECM) components, consistently with their different degree of differentiation (Masters et al, 1986; Nista et al, 1996). After infection and G418 selection, the productive insertion of the IL-2 gene was evaluated by mRNA analysis in RT-PCR. As shown in Figure 1, neither parental (RT112– and EJ–wt) nor control vector-infected (RT112– and EJ–neo) cells produced IL-2-specific PCR products, while IL-2-transduced (RT112– and EJ–IL-2) cells produced a consistent amount of IL-2 mRNA. Moreover, both RT112–IL-2 and EJ–IL-2 showed consistent release of biologically active IL-2 (194 and 113 IU/$10^6$ cells/48 h, respectively) that remained practically unchanged during a period of over 6 months. Both IL-2 mRNA expression and protein production by transfected cells were stable upon several freeze-thawing cycles and detectable amounts of IL-2 were released for at least 2 weeks after $\gamma$-irradiation with up to 10 000 cGy (data not shown).

### Phenotypic and functional characterization of IL-2-transduced human bladder cancer cell lines

Expression of potentially functional IL-2 receptors (IL-2R), as well as IL-2-induced functional modifications, has been recently described on cells of non-lymphoid origin (Velotti et al, 1994; Yasumura et al, 1994). Although not conclusive, evidence of the expression of IL-2R $\alpha$, $\beta$, and $\gamma$ chains has been reported also for RT112 and EJ cell lines (Velotti et al, 1994). These findings raise the question whether constitutive IL-2 production might modify the phenotypic and functional characteristics of the transduced cells; thus, we initially tested parental and IL-2-transduced RT112 and EJ cells for morphology, in vitro growth rate, and [$^3$H]TdR incorporation (Figure 2). No differences were found in RT112–IL-2 or EJ–IL-2 as compared to their parental or control vector-transduced counterparts. Even in the presence of absolute or relative growth factor deprivation, no significant changes in proliferation rate were seen (Figure 2B).

We then investigated whether IL-2 transduction alters the pattern of other cytokine genes expressed by RT112 and EJ cells. As shown in Figure 3, the expression of IL-6, TNF-$\alpha$, and TGF-$\beta$ mRNAs, which are constitutively expressed in both cell lines, remained unmodified in IL-2-transduced cells in comparison to control vector-infected cells. Similarly, GM-CSF and IL-4 mRNAs, which were poorly expressed or absent in control vector-infected cells, were not induced following IL-2 gene transduction.

Immunofluorescence and cytofluorimetric analysis showed no significant difference in the expression of either HLA class I or several surface adhesion molecules involved in the control of tumour–immune cell interactions, as well as tumour growth and invasion, such as $\beta$1 integrins, $\alpha 6\beta 4$, $\alpha v\beta 3$, ICAM-1, CD44 with its alternatively spliced variants v4, v6, and v9, between RT112–IL-2 or EJ–IL-2 and their parental or control vector-transduced counterparts (Figure 4 and Figure 5, respectively).

### Tumorigenicity into *nu/nu* mice of IL-2-transduced human bladder cancer cell lines

The ability of both RT112 and EJ human bladder cancer cell lines to grow and form established tumours when injected subcutaneously into *nu/nu* mice has been previously reported (Masters et al, 1986). To assess whether IL-2 gene transduction affects their tumorigenicity, *nu/nu* mice were challenged with progressive doses of either control or IL-2 gene-transduced RT112 or EJ cells. As shown in Table 1, none of the mice challenged with RT112–IL-2 or EJ–IL-2 developed palpable tumours over an observation period of more than 200 days; conversely, the majority of RT112–neo- and EJ–neo-injected mice developed progressively growing tumours with latency and tumour growth times inversely proportional to the number of challenging tumour cells.

To evaluate the importance of the distinct *nu/nu* host effector cell populations potentially involved in the rejection of the IL-2-transduced or control-vector-infected EJ cells, we performed a second series of experiments in which neutrophils, natural killer (NK) cells and radiosensitive cells had been depleted in vivo prior to the tumour challenge. All of the mice challenged with $10^7$ EJ–neo cells

 © Cancer Research Campaign 1999

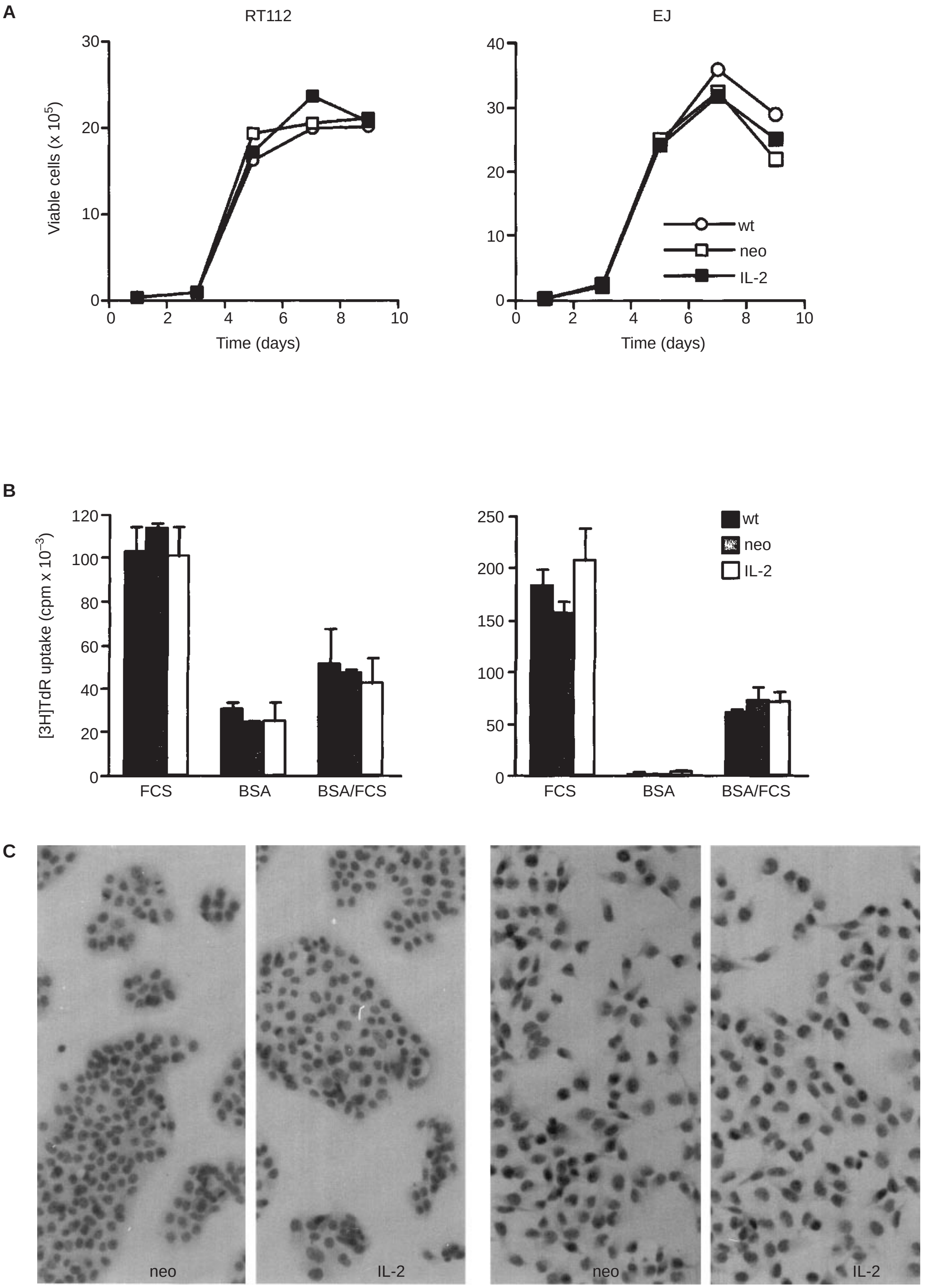

**Figure 2** In vitro growth, $[^3H]$TdR uptake, and morphology of IL-2-transduced RT112 and EJ human bladder carcinoma cell lines. Parental (wt), control-vector (neo), and IL-2 (IL-2)-transduced RT112 and EJ cells were analysed for in vitro growth, $[^3H]$TdR uptake and morphology. In vitro growth was evaluated by seeding $0.5 \times 10^5$ cells/well in a 6-well tissue culture plate in standard medium containing 10% FCS and by counting the number of viable cells under light microscopy every 48 h for up to 9 days (**A**). Proliferative ability was evaluated by measuring $[^3H]$TdR uptake after 96 h in different culture conditions: 96 h in standard medium containing 10% FCS, 96 h in medium containing 0.5% BSA, or 48 h in medium containing 0.5% BSA followed by 48 h in medium containing 5% FCS. Cells were then pulsed with $[^3H]$TdR for 18 h, harvested, and the $[^3H]$TdR uptake was measured by liquid scintillation counting. Experiments were performed in triplicates and results are expressed as average cpm ± SE (**B**). Results are representative of one out of four independent experiments. Morphology was evaluated by culturing the cells on tissue-culture-treated glass slides; cells were then fixed and stained with haematoxylin and eosin, and microphotographs were taken at × 400 (**C**)

*© Cancer Research Campaign 1999*

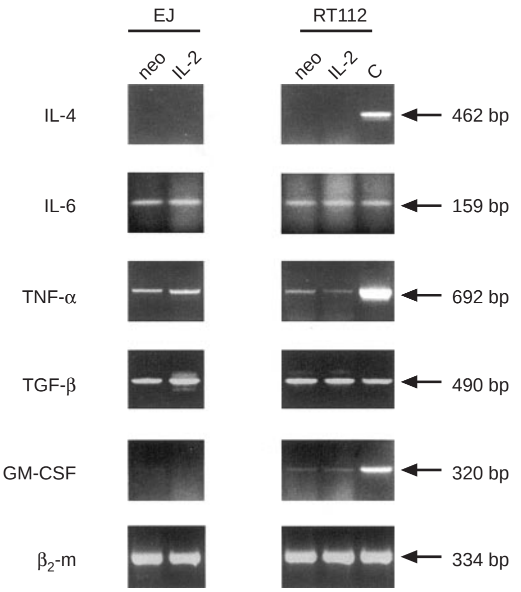

**Figure 3** Cytokine mRNA expression by IL-2-transduced RT112 and EJ human bladder carcinoma cell lines. Control-vector (neo) and IL-2 (IL-2)-transduced RT112 and EJ cells were analysed for cytokine mRNAs by RT-PCR. Total cellular RNA was extracted and reverse transcription, as well as cDNA amplification, was performed as described in Materials and methods. RNAs from T-cell blasts obtained by 72 h-PHA stimulation of human PBMC from healthy donors (IL-4, TNFα, TGFβ, and GM-CSF) or RPMI 8866 EBV$^+$ B lymphoblastoid cell line (IL-6) were used as positive controls (C). The $\beta_2$-m amplification represents the positive control of cDNA transcription developed progressively growing tumours, but both latency and tumour growth times were significantly shorter in polymorphonuclear (PMN) and NK cell-depleted animals than in untreated or irradiated mice (Table 2). With regard to EJ–IL-2 cells, the tumour challenge was completely rejected by all mice that remained tumour free for up to 200 days regardless of the immunosuppressive treatment received, although the maximum tumour diameter reached before rejection was significantly higher in mice deprived of PMN cells and in those receiving irradiation (Table 3).

### DISCUSSION

In this study we provide evidence that the human IL-2 gene can be stably and productively transduced into human bladder cancer cells without modifying their phenotypic and functional features. The constitutive IL-2 release abrogates human transitional bladder cancer ability to grow into *nu*/*nu* mice, by eliciting in the immunosuppressed host an anti-tumour activity based on different effector cell populations.

Retroviral vector-mediated stable and productive insertion of the IL-2 gene into human cancer cells has been documented for several tumours, such as melanoma, renal cell carcinoma, myeloid and lymphoid leukaemic cells, colon carcinoma, and lung carcinoma (Gansbacher et al, 1992; Gastl et al, 1992; Cignetti et al, 1994; Guarini et al, 1996; Stein et al, 1996). With regard to urothelial tumours, adenoviral-mediated gene transfer of both the reporter gene luciferase and a truncated form of the oncosuppressive gene RB-1 have been reported (Bass et al, 1995; Xu et al, 1996). The present study provides the first evidence that human transitional cell carcinoma of the bladder can be engineered to constitutively secrete biologically active human IL-2 by using retroviral vectors. In many instances, living and proliferating cancer cells appear to be more efficient as a vaccine than those whose proliferative ability is blocked by irradiation or mitomycin C treatment. Nevertheless, the possibility to introduce undesired variables in engineered cancer cell behaviour is still of major concern in view of their clinical use, precluding the development of live cancer vaccines. With regard to bladder carcinoma, the possibility that certain cytokines, namely IL-2 and IL-6, may act as autocrine growth factors has been reported (Hawkyard et al, 1993; Yasumura et al, 1994; Okamoto et al, 1997). Moreover, a previous study from our group (Velotti et al, 1994) has shown that mRNA for the α chain of the IL-2R is expressed by both RT112 and EJ cell lines, whereas mRNA for the γ chain is clearly evident in the RT112 cell line and detectable only after a long exposure in the EJ cell line; with regard to the IL-2R β chain, both cell lines positively stain with anti-p70/75 mAbs, although neither express specific mRNA sequences, as assessed by PCR. Furthermore, recent results from our group indicate that no modulation of IL-2R expression, at both the mRNA and protein level, is observed in IL-2 gene-transduced RT112 and EJ cells, as compared to their control-vector-transfected or parental counterparts (F Velotti, unpublished observations). The extensive functional and phenotypic characterization performed in the present study suggests that transduction of the human bladder cancer cell lines RT112 and EJ with the IL-2 gene does not affect their morphology, growth rate or proliferation, nor does it modify the pattern of other cytokine gene or membrane adhesion receptor, as well as HLA class I expression. These results are in agreement with previous studies of IL-2 gene transfer in human renal carcinoma, small cell lung carcinoma and leukaemia cells (Gastl et al, 1992; Cignetti et al, 1994; Meazza et al, 1996). However, some alterations in tumour cell behaviour and phenotype following IL-2 gene transduction in human cancer cells have been recently described. IL-2-transduced renal carcinoma cell lines exhibit an enhanced ICAM-1 and a reduced CD44 expression, as well as a decreased adhesiveness to ECM components, as compared with their parental counterparts (Hathorn et al, 1994); TGF-β1 down-modulation has been documented in both renal cell carcinoma and lung adenocarcinoma following IL-2 gene transfer (Hathorn et al, 1994; Guarini et al, 1996); decreased mdr-1 expression and increased chemosensitivity has been shown in IL-2 gene-transduced human colon carcinoma cell lines (Stein et al, 1996).

As reported for several IL-2-transduced human tumours of different histologic origin (Gansbacher et al, 1992; Gastl et al, 1992; Cignetti et al, 1994; Guarini et al, 1996), bladder carcinoma cells engineered to release IL-2 lose their tumorigenic ability in immunosuppressed mice. Our results using selective immunodepletion techniques suggest that multiple cell populations are involved in the rejection of IL-2-releasing cells, among which a prominent role appears to be played by PMN and other radiosensitive cells. Polymorphonuclear granulocyte recruitment and activation has been implicated as a pivotal effector mechanism during rejection responses elicited by IL-2-engineered cancer cells (Cavallo et al, 1992; Colombo et al, 1992; Meazza et al, 1996; Musiani et al, 1997). In both immunocompetent mice challenged with an IL-2-secreting syngeneic mammary adenocarcinoma (Cavallo et al, 1992) and athymic mice challenged with an IL-2 gene-transduced human small cell lung carcinoma (Meazza et al, 1996), the appearance of a neutrophil infiltrate at the periphery of the tumour cell aggregate is an early (days 3 and 2 after challenge, respectively) event in the rejection process, suggesting that the

*© Cancer Research Campaign 1999*

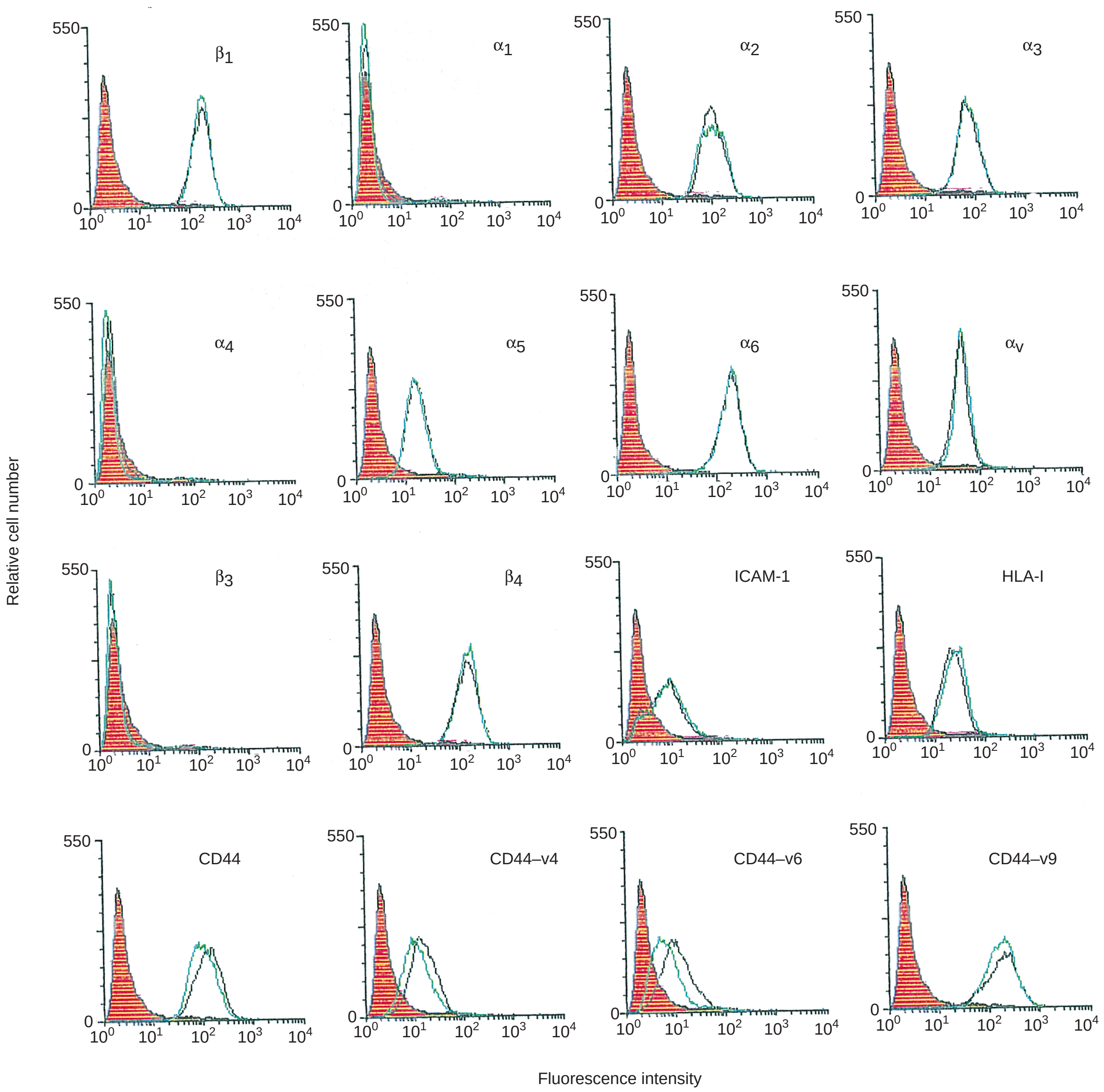

**Figure 4** Cytofluorimetric analysis of HLA class I and adhesion molecule expression on control-vector or IL-2-transfected RT112 cells. RT112–neo (black profile) and RT112–IL-2 (green profile) were stained with specific mAbs (see Materials and methods) and a fluorescein-conjugated secondary Ab. The red area represents negative control obtained by staining cells with an isotype-matched irrelevant mAb presence of tumour neovascularization is not required. In the former model, moreover, the extent and rapidity of neutrophil recruitment is proportional to the amount of IL-2 released from the transduced cells. IL-2 may recruit and activate PMN cells either directly through the interaction with its receptor (Djeu et al, 1993; Liu et al, 1994), leading to cytokine secretion and stimulation of tumoricidal activity (Wei et al, 1993, 1994; Girard et al, 1995; Pericle et al, 1996), or through its action on T- and NK cells, leading to a cascade of secondary neutrophil-activating cytokines and chemokines, such as IL-8 and TNF-α.

The subsequent complete rejection of EJ–IL-2 cells by PMN-depleted and irradiated *nu*/*nu* mice suggests the emergence of alternative effector mechanisms, mediated by NK cells and monocyte/macrophages. Both NK cells and monocyte/macrophages have indeed been implicated in the rejection of IL-2-secreting cancer cells by mice lacking functional T- and B-cells (Alosco et al, 1993; Hara et al, 1995). Moreover, as shown in Table 2, NK cells, as well as radioresistant populations, play a relevant role in the growth control of xenografted EJ–neo cells.

The recent work by Meazza and colleagues (Meazza et al, 1996) on IL-2-transduced small cell lung carcinoma suggests that, in addition to the activation of macrophages (Verstovsek et al, 1992), IL-2 may exert its effects by altering tumour vascularization either by a direct action on endothelial cells (Hicks et al, 1991), or by promoting a cascade of secondary cytokines (such as IL-1, TNF-α, IL-8), which in turn mediate further vascular and tissue damage, and amplify neutrophil recruitment. Although the role of such mechanisms in the rejection of IL-2 gene-transduced

*© Cancer Research Campaign 1999*

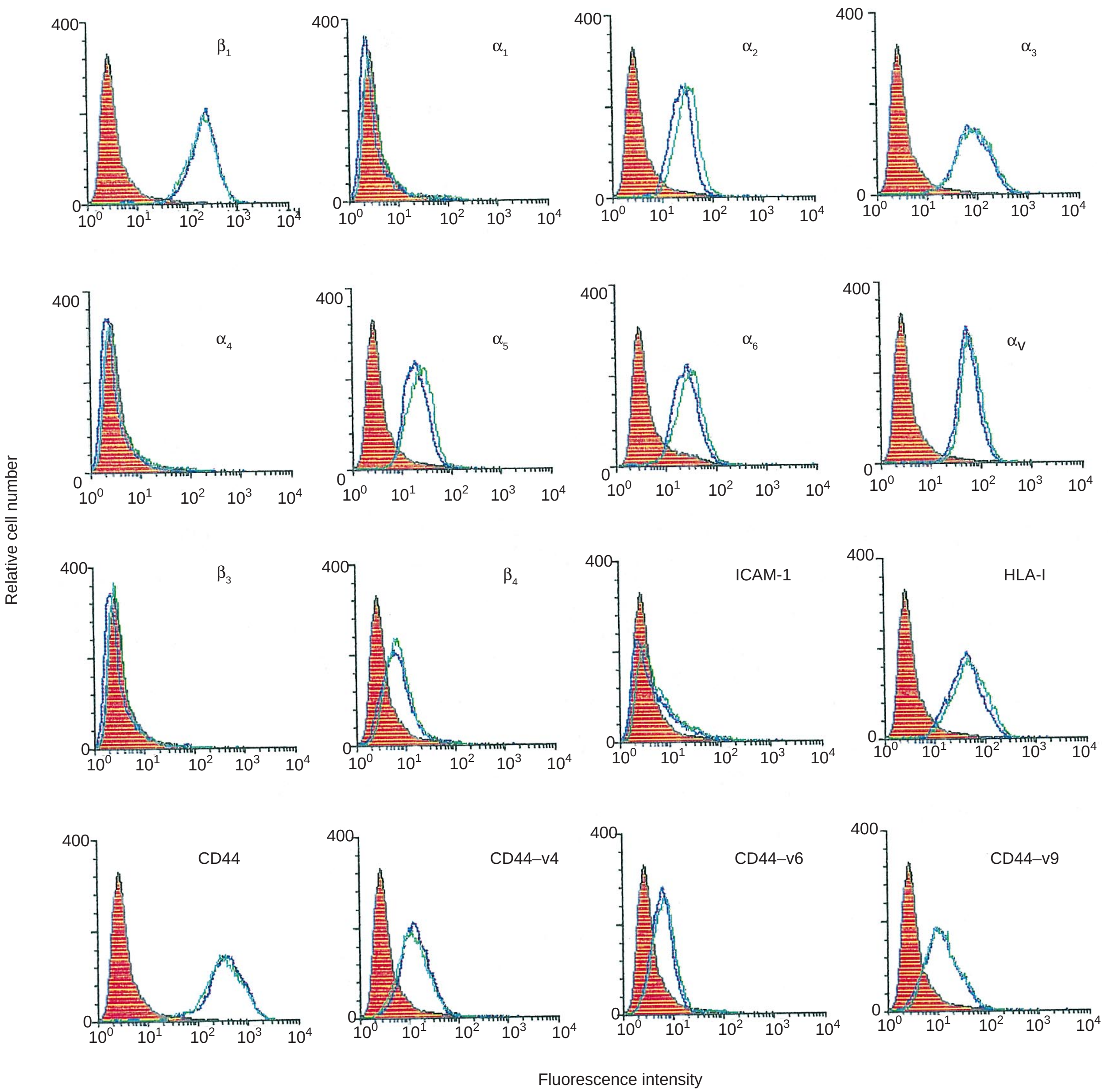

**Figure 5** Cytofluorimetric analysis of HLA class I and adhesion molecule expression on control-vector or IL-2-transfected EJ cells. EJ–neo (blue profile) and EJ–IL-2 (green profile) were stained with specific mAbs (see Materials and methods) and a fluorescein-conjugated secondary Ab. The red area represents negative control obtained by staining cells with an isotype-matched irrelevant mAb bladder cancer cells has not been explored in the present study, an even more interesting support to their potential relevance in this tumour model comes from our earlier experience with intra-arterial or intravesical rIL-2 treatment in patients with superficial transitional cell carcinoma of the bladder (Tubaro et al, 1991, 1995; Velotti et al, 1991*a*, 1991*b*). In these clinical trials we observed a significant increase in the inflammatory response at the tumour site, with evident infiltration of tumour stroma and neoplastic epithelium by activated ($CD25^+$/$HLA\text{-}DR^+$) T-cells, macrophages and both eosinophil and PMN. Increase in tumour-associated inflammatory response appeared to be a necessary step for T-cell activation and IFN-$\gamma$ release, which in turn induced IL-1$\alpha$, IL-1$\beta$ and TNF-$\alpha$ production from macrophages. Moreover, increase of tumour-infiltrating leucocytes and their cytokine secretion were associated with the expression of adhesion molecules (ICAM-1, VCAM-1 and E-selectin) on the vascular endothelium.

Unfortunately, studies with human tumours in *nu*/*nu* mice do not allow for evaluating the ability of cytokine-transduced cells to elicit a T-cell response. In vitro experiments with IL-2-engineered human melanoma and lung carcinoma cells cocultured with allogeneic or autologous peripheral blood mononuclear cells (PBMC) have shown that IL-2 released by the engineered cells is capable of supporting $CD3^+$ and, to a lesser extent, $CD56^+$ cell viability, and to stimulate both promiscuous and specific cytotoxic activity (Guarini et al, 1995, 1997). Preliminary results with RT112– and EJ–IL-2 cells cocultured with PBMC from partially HLA-matched donors suggest that they are capable of stimulating T-cell growth

© Cancer Research Campaign 1999

**Table 1** Tumorigenicity in *nu/nu* mice of the IL-2-transduced human bladder carcinoma cell lines RT112 and EJ

| Cell line | Challenge (no. of cells) | Tumour take[a] | Latency time[b] (days) | Tumour growth time[c] (days) |
|---|---|---|---|---|
| RT112–neo | $1 \times 10^6$ | 7/8 | 24.2 ± 3.8 | 44.9 ± 4.4 |
| | $5 \times 10^6$ | 7/8 | 17.9 ± 3.1 | 30.8 ± 3.8 |
| | $10 \times 10^6$ | 8/8 | 18.3 ± 4.2 | 29.3 ± 4.1 |
| RT112–IL-2 | $1 \times 10^6$ | 0/8 | – | > 200 |
| | $5 \times 10^6$ | 0/8 | – | > 200 |
| | $10 \times 10^6$ | 0/8 | – | > 200 |
| EJ–neo | $1 \times 10^6$ | 8/8 | 18.2 ± 3.7 | 40.3 ± 9.1 |
| | $5 \times 10^6$ | 8/8 | 15.7 ± 4.1 | 35.9 ± 5.3 |
| | $10 \times 10^6$ | 8/8 | 14.4 ± 4.2 | 28.6 ± 6.8 |
| EJ–IL-2 | $1 \times 10^6$ | 0/8 | – | > 200 |
| | $5 \times 10^6$ | 0/8 | – | > 200 |
| | $10 \times 10^6$ | 0/8 | – | > 200 |

[a]Tumour take: No. of mice developing a palpable tumour/No. of mice challenged. Data from two separate experiments have been cumulated. [b]Latency time: Days from the challenge to the development of a tumour with a mean diameter of 3 mm (average ± SE). [c]Tumour growth time: Days from the challenge to the development of a tumour with a mean diameter of 10 mm (average ± SE).

**Table 2** Tumorigenicity of EJ–neo cells in immunodepleted *nu/nu* mice

| Treatment | Tumour take[a] | Latency time[b] (days) | *P*[c] | Tumour growth time[d] (days) | *P* |
|---|---|---|---|---|---|
| None | 12/12 | 15.3 ± 4.7 | | 59.8 ± 7.6 | |
| α- PMN | 10/10 | 10.1 ± 1.7 | 0.003 | 51.0 ± 3.8 | 0.004 |
| α-asialo $GM_1$ | 10/10 | 09.9 ± 1.7 | 0.002 | 45.5 ± 6.9 | < 0.001 |
| Irradiation | 10/10 | 13.8 ± 1.6 | NS | 53.1 ± 9.8 | 0.088 |
| Irradiation + α-asialo $GM_1$ | 10/10 | 10.3 ± 1.8 | 0.004 | 46.8 ± 5.2 | < 0.001 |

Mice were treated as described in Materials and methods and challenged s.c. with $10^7$ EJ–neo cells. [a]Tumour take: No. of mice developing a palpable tumour/No. of mice challenged. Data from two separate experiments have been cumulated. [b]Latency time: Days from the challenge to the development of a tumour with a mean diameter of 3 mm (average ± SE). [c]*P*-value is calculated by comparing mean latency or tumour growth times between each treatment group and untreated controls by 'two-tailed student's t-test'. [d]Tumour growth time: Days from the challenge to the development of a tumour with a mean diameter of 10 mm (average ± SE).

**Table 3** Maximum tumour diameter reached by EJ–IL-2 cells before rejection in immunodepleted *nu/nu* mice

| | Tumour size (mm)[a] | | |
|---|---|---|---|
| Treatment | Distribution | Mean ± SE | *P*[b] |
| None | 0, 0, 0, 0, 1, 1, 1, 1, 1, 2 | 0.7 ± 0.7 | |
| α-PMN | 1, 1, 2, 2, 2, 2, 2, 2, 3, 3 | 2.0 ± 0.7 | < 0.001 |
| α-asialo $GM_1$ | 0, 0, 0, 1, 1, 1, 1, 2, 2, 2 | 1.0 ± 0.8 | NS |
| Irradiation | 1, 1, 2, 2, 2, 2, 2, 2, 3, 3 | 2.0 ± 0.7 | < 0.001 |
| Irradiation + α-asialo $GM_1$ | 1, 1, 1, 2, 2, 2, 2, 2, 2, 3 | 1.8 ± 0.6 | 0.001 |

[a]Mice were treated as described in Materials and methods and challenged s.c. with $10^7$ EJ–IL-2 cells. Maximal tumour growth was reached by day 11 after challenge. All mice completely rejected the tumour by day 34 and remained tumour-free for up to 200 days. Data from two separate experiments have been cumulated. [b]*P*-value is calculated by comparing mean tumour size between each treatment group and untreated controls by 'two-tailed Student's *t*-test'.

and lymphokine activated killer-like cytotoxicity (M Milella, unpublished results). Moreover, the recent work from one of us (Guarini et al, 1997) provides evidence that IL-2 gene transfer can overcome tumour-induced immunosuppression much more efficiently than rIL-2.

Although further studies are needed, especially in an attempt to identify molecular targets for a specific immune intervention against human transitional cell carcinoma of the bladder, the findings reported in the present study offer a valuable support to the development of immunization strategies employing bladder carcinoma cells, ex vivo transduced with the IL-2 gene.

### ACKNOWLEDGEMENTS

This work was partially supported by grants from Associazione Italiana per la Ricerca sul Cancro (AIRC) and Ministero della Sanità. We wish to thank P Birarelli, AM Bressan, D Milana, A Procaccini and A Sabatucci for their expert technical assistance.

© Cancer Research Campaign 1999

### REFERENCES

Alosco T, Croy BA, Gansbacher B, Wang HQ, Rao U and Bankert R (1993) Anti-tumour response independent of functional B and T lymphocytes induced by the local and sustained release of interleukin-2 by the tumour cell. *Cancer Immunol Immunother* **36**: 364–372

Bass C, Cabrera G, Elgavish A, Robert B, Siegal GP, Anderson SC, Maneval DC and Curiel DT (1995) Recombinant adenovirus-mediated gene transfer to genitourinary epithelium in vitro and in vivo. *Cancer Gene Ther* **2**: 97–104

Cavallo F, Giovarelli M, Gulino A, Vacca A, Stoppacciaro A, Modesti A and Forni G (1992) Role of neutrophils and CD4+ T lymphocytes in the primary and memory response to non-immunogenic murine mammary adenocarcinoma made immunogenic by IL-2 gene. *J Immunol* **149**: 3627–3635

Chomczynski P and Sacchi N (1987) Single-step method of RNA isolation by acid guanidinium thiocyanate-phenol-chloroform extraction. *Anal Biochem* **162**: 156–159

Cignetti A, Guarini A, Carbone A, Forni M, Cronin K, Forni G, Gansbacher B and Foà R (1994) Transduction of the IL-2 gene into human acute leukemia cells: induction of tumor rejection without modifying cell proliferation and IL-2 expression. *J Natl Cancer Inst* **86**: 785–791

Colombo MP, Modesti A, Parmiani G and Forni G (1992) Local cytokine availability elicits tumor rejection and systemic immunity through granulocyte-T-lymphocyte cross-talk. *Cancer Res* **52**: 4853–4857

Connor J, Bannerji R, Saito S, Heston W, Fair W and Gilboa E (1993) Regression of bladder tumours in mice treated with IL-2 gene-modified tumour cells. *J Exp Med* **177**: 1127–1134

Djeu JY, Liu JH, Wei S, Rui H, Pearson CA, Leonard WJ and Blanchard DK (1993) Function associated with IL-2 receptor-beta on human neutrophils. Mechanism of activation of antifungal activity against *Candida albicans* by IL-2. *J Immunol* **150**: 960–70

Dranoff G and Mulligan RC (1995) Gene transfer as cancer therapy. *Adv Immunol* **58**: 417–454

Dusty Miller A and Rosman GJ (1989) Improved retroviral vectors for gene transfer and expression. *Biotechniques* **7**: 980–986

Fearon ER, Pardoll DM, Itaya T, Golumbek P, Levitsky HI, Simons JW, Karasuyama H, Vogelstein B and Frost P (1990) Interleukin-2 production by tumour cells bypasses T helper function in the generation of an anti-tumour response. *Cell* **60**: 397–403

Forni G, Cavallo GP, Giovarelli M, Benetton G, Jemma C, Barioglio MG, De Stefani A, Forni M, Santoni A, Modesti A, Cavallo G, Menzio P and Cortesina G (1988) Tumor immunotherapy by local injection of interleukin-2 and non-reactive lymphocytes. Experimental and clinical results. *Prog Exp Tumor Res* **32**: 187–212

Gansbacher B, Zier K, Cronin K, Hantzopoulos PA, Bouchard B, Houghton A, Gilboa E and Golde D (1992) Retroviral gene transfer induced constitutive expression of interleukin-2 or interferon-γ in irradiated human melanoma cells. *Blood* **80**: 2817–2825

Gastl G, Finstad CL, Guarini A, Golde D, Bosl G, Bander NH, Gilboa E and Gansbacher B (1992) Retroviral vector-mediated lymphokine gene transfer into human renal cancer cells. *Cancer Res* **52**: 6229–6236

Girard D, Gosselin J, Heitz D, Paquin R and Beaulieu AD (1995) Effects of interleukin-2 on gene expression in human neutrophils. *Blood* **86**: 1170–1176

Guarini A, Gansbacher B, Cronin K, Fierro MT and Foà R (1995) IL-2 gene-transduced human HLA-A2 melanoma cells can generate a specific anti-tumour cytotoxic T-lymphocyte response. *Cytokines Mol Ther* **1**: 57–64

Guarini A, Riera L, Reato G, Carbone A, Cignetti A, Gillio Tos A, Lanfrancone L, Melani C, Paul RW, Forni G and Foà R (1996) Human lung carcinoma cells engineered to release IL-2, IL-7, GM-CSF, and TNF-α: growth in nu/nu mice and modulation of TGF-β 1 production. *Int J Oncol* **8**: 765–772

Guarini A, Riera L, Cignetti A, Montacchini L, Massaia M and Foà R (1997) Transfer of the interleukin-2 gene into human cancer cells induces specific anti-tumour recognition and restores the expression of CD3/T-cell receptor associated signal transduction molecules. *Blood* **89**: 212–218

Hara I, Nguyen H, Takechi Y, Gansbacher B, Chapman PB and Houghton AN (1995) Rejection of mouse melanoma elicited by local secretion of interleukin-2: implicating macrophages without T cells or natural killer cells in tumor rejection. *Int J Cancer* **61**: 253–260

Hathorn RW, Tso CL, Kaboo R, Pang S, Figlin R, Sawyers C, deKernion JB and Belldegrun A (1994) In vitro modulation of the invasive and metastatic potentials of human renal cell carcinoma by interleukin-2 and/or interferon-α gene transfer. *Cancer* **74**: 1904–1911

Hawkyard SJ, Jackson AM, Prescott KJ and Chisholm GD (1993) The effect of recombinant cytokines on bladder cancer cells in vitro. *J Urol* **150**: 514–518

Hicks C, Cooley MA and Penny R (1991) Investigation of interleukin-2 receptors on human endothelial cells. *Growth Factors* **5**: 201–208

Liu JH, Wei S, Ussery D, Epling-Burnette PK, Leonard WJ and Djeu JY (1994) Expression of interleukin-2 receptor gamma chain on human neutrophils. *Blood* **84**: 3870–3875

Lum BL and Torti FM (1995) Immunotherapy and the prospects for immunologic-based gene therapy in superficial bladder cancer. *FORUM Trends Exp Clin Med* **5**: 410–439

Masters JRW, Hepburn PJ, Walker L, Highman WJ, Trejdosiewicz LK, Povey S, Parkar M, Hill BT, Riddle PR and Franks LM (1986) Tissue culture model of transitional cell carcinoma: characterization of twenty-two human urothelial cell lines. *Cancer Res* **46**: 3630–3636

Meazza R, Marciano S, Sforzini S, Orengo AM, Coppolecchia M, Musiani P, Ardizzoni A, Santi L, Azzarone B and Ferrini S (1996) Analysis of IL-2 receptor expression and of the biological effects of IL-2 gene transfection in small cell lung cancer. *Br J Cancer* **74**: 788–795

Melani C, Chiodoni C, Arienti F, Maccalli C, Sule-Suso J, Anichini A, Colombo MP and Parmiani G (1994) Cytokine gene transduction in tumour cells: IL-2 or IL-4 gene transfer in human melanoma cells. *Nat Immun Cell Growth Regul* **13**: 76–94

Musiani P, Modesti A, Giovarelli M, Cavallo F, Colombo MP, Lollini PL and Forni G (1997) Cytokines, tumour-cell death and immunogenicity: a question of choice. *Immunol Today* **18**: 32–36

Nista A, Mattioni M, Gismondi A, Palmieri G and Santoni A (1996) β1 integrin expression and function in human bladder cancer cells: modulation by TNFα. *Anticancer Res* **16**: 581–588

Okamoto M, Hattori K and Oyasu R (1997) Interleukin-6 functions as an autocrine growth factor in human bladder carcinoma cell lines in vitro. *Int J Cancer* **72**: 149–194

Pericle F, Kirken RA, Epling-Burnette PK, Blanchard DK and Djeu JY (1996) Direct killing of interleukin-2-transfected tumor cells by human neutrophils. *Int J Cancer* **66**: 367–373

Sette A, Adorini L, Marubini E and Doria G (1986) A microcomputer program for probit analysis of interleukin-2 (IL-2) titration data. *J Immunol Methods* **75**: 34–40

Sitkovsky MV and Paul WE (1988) Immunology. Global or directed exocytosis? *Nature* **332**: 306–307

Stein U, Walther W and Shoemaker RH (1996) Reversal of multidrug resistance by transduction of cytokine genes into human colon carcinoma cells. *J Natl Cancer Inst* **88**: 1383–1392

Tubaro A, Velotti F, Stoppacciaro A, Santoni A, Vicentini C, Bossola PC, Galassi P, Pettinato A, Morrone S, Napolitano T, Frati L, Ruco L, Franks CR, Palmer PA, Pourreau CN and Miano L (1991) Continuous intra-arterial administration of recombinant interleukin-2 in low-stage bladder cancer. *Cancer* **68**: 56–61

Tubaro A, Stoppacciaro A, Velotti F, Bossola PC, Cusumano G, Vicentini C, De Carli P, Ruco L, Santoni A, Cancrini A, Giuffrida AM, Cippitelli M, Palmer PA and Miano L (1995) Local immunotherapy of superficial bladder cancer by intravesical instillation of recombinant interleukin-2. *Eur Urol* **28**: 297–303

Velotti F, Stoppacciaro A, Ruco L, Tubaro A, Pettinato A, Morrone S, Napolitano T, Bossola PC, Franks CR, Palmer PA, Pourreau CN, Piccoli M, Baroni CD, Frati L, Miano L and Santoni A (1991*a*) Local activation of immune response in bladder cancer patients treated with intra-arterial infusion of recombinant interleukin-2. *Cancer Res* **51**: 2456–2462

Velotti F, Stoppacciaro A, Tubaro A, Giuffrida A, Morrone S, Ruco L, Vicentini C, Tonelli VH, Miano L and Santoni A (1991*b*) Immune response in bladder cancer patients (pts) treated with two different recombinant interleukin-2 (rIL-2) local/regional regimens. *Proc Am Ass Cancer Res* (abstract 246)

Velotti F, Cippitelli M, Giuffrida AM, Punturieri A, Moretti F, Stoppacciaro A, Tubaro A, De Maria R and Santoni A (1994) Local immunotherapy of human bladder cancer with recombinant IL-2: activation of immune response and possible direct action of IL-2 on tumour cells. In *Cytokine-induced Tumour Immunogenicity. From Exogenous Molecules to Gene Therapy*, Forni G, Foà R, Santoni A, Frati L (eds), pp. 429–422 Academic Press: London

Verstovsek S, MacCubbin D, Ehrka MJ and Mihich E (1992) Tumoricidal activation of murine resident peritoneal macrophages by interleukin-2 and tumor necrosis factor α. *Cancer Res* **52**: 3880–3885

Xu H, Zhou Y, Seigne J, Perng G, Mixon M, Zhang C, Li J, Benedict WF and Hu S (1996) Enhanced tumour suppressor gene therapy via replication-deficient adenovirus vectors expressing an N-terminal truncated retinoblastoma protein. *Cancer Res* **56**: 2245–2249

 © Cancer Research Campaign 1999

Yasumura S, Lin WC, Weidmann E, Hebda P and Whiteside TL (1994) Expression of interleukin-2 receptors on human carcinoma cell lines and tumour growth inhibition by interleukin-2. *Int J Cancer* **59**: 225–234

Wei S, Blanchard DK, Liu JH, Leonard WJ and Djeu JY (1993) Activation of tumor necrosis factor-alpha production from human neutrophils by IL-2 via IL-2-R beta. *J Immunol* **150**: 1979–1987

Wei S, Liu JH, Blanchard DK and Djeu JY (1994) Induction of IL-8 gene expression in human polymorphonuclear neutrophils by recombinant IL-2. *J Immunol* **152**: 3630–3636

*© Cancer Research Campaign 1999*
